# Taking a Low Glycemic Index Multi-Nutrient Supplement as Breakfast Improves Glycemic Control in Patients with Type 2 Diabetes Mellitus: A Randomized Controlled Trial

**DOI:** 10.3390/nu6125740

**Published:** 2014-12-10

**Authors:** Di Li, Peiwen Zhang, Honghui Guo, Wenhua Ling

**Affiliations:** 1 Guangdong Provincial Key Laboratory of Food, Nutrition and Health, Department of Nutrition, School of Public Health, Sun Yat-Sen University (Northern Campus), Guangzhou 510080, China; E-Mails: danyinghm@hotmail.com (D.L.); zhangpeiwen0719@163.com (P.Z.); 2 Department of Nutrition, Henry Fok School of Food Science and Engineering, Shaoguan University, Shaoguan 512005, China

**Keywords:** breakfast replacement, glycemic control, low glycemic index, multi-nutrient supplement, type 2 diabetes mellitus

## Abstract

Dietary therapy is the mainstay of treatment for diabetes. This study examined the effect of a low glycemic index (GI) multi-nutrient supplement, consumed in place of breakfast, on glycemic control in patients with type 2 diabetes mellitus (T2DM). A total of 71 participants were randomized at a 2:1 ratio into either a breakfast replacement group or a normal breakfast group for a 12-week interventional study. The primary outcome measure was change in hemoglobin A1c (HbA1c). Nutrition status and somatometry were studied as secondary outcomes. The breakfast replacement group displayed a −0.2% absolute reduction in HbA1c (95% CI (confidence interval), −0.38% to −0.07%, *p* = 0.004), while the HbA1c of the control group increased 0.3% (95% CI, 0.1% to 0.5%, *p* = 0.005). The baseline Mini Nutritional Assessment score for both groups was 26.0 and no significant changes occurred following intervention. However, there was a statistically significant difference in body mass index between the treatment and control groups (*p* = 0.032) due to the weight gain in the control group (increased 0.5 kg, 95% CI was 0.2 to 0.9, *p* = 0.007). These data suggest that breakfast replacement with a low GI multi-nutrient supplement can improve glycemic and weight control in T2DM.

## 1. Introduction

Type 2 diabetes mellitus (T2DM) is a chronic progressive metabolic disorder characterized by hyperglycemia and glucose intolerance [1]. It leads to numerous and varied complications, such as in the cardiovascular, nervous and urinary systems, and is the seventh leading cause of death in 2010 according to the World Health Organization [2]. Alarmingly, the incidence of T2DM has almost doubled during the past three decades [3,4]. In China, there is also a widespread occurrence of T2DM with an overall prevalence estimated by a report published in 2013 at 11.6% of the total population, 12.1% of men and 11.0% of women [5]. Therefore, strategies to prevent and treat diabetes are urgently needed.

Currently, hyperglycemia is thought to be effectively controlled by medication and lifestyle interventions. In terms of lifestyle, changes in patient diet play an important role in preventing and treating T2DM [6]. Numerous studies on lifestyle interventions have observed that a low glycemic index (GI) diet helped control glycemia not only in T2DM patients, but also in healthy people [7,8,9]. However, low-GI diet may not be meeting other nutrient targets, which is not conducive to the nutrition status and health of T2DM patients [10].

With the aim to determine a simple and safe way to control blood glucose in T2DM patients, we conducted an intervention study using a low GI multi-nutrient supplement powder that was primarily made of rice, soybean, oat dietary fiber, bitter gourd, multi-vitamins and minerals, which was consumed in place of breakfast by T2DM patients. Our results suggest this may help control T2DM.

## 2. Experimental Section

### 2.1. Participants

**Figure 1:**
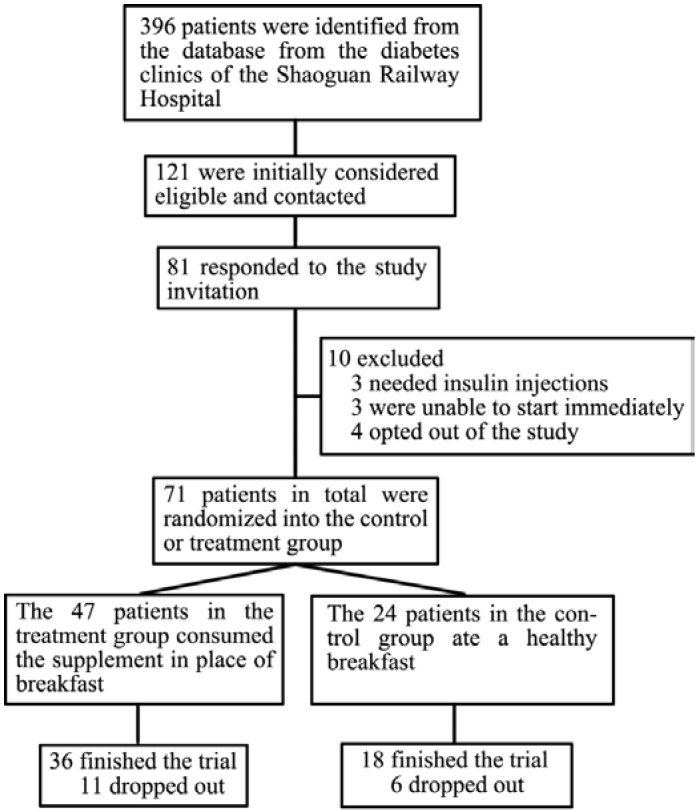
Flow diagram of recruitment.

Potential participants were identified from the diabetes clinic database of Shaoguan Railway Hospital (Shaoguan, China). 81 of all the 396 patients in the database responded to the study invitation while 10 were excluded (3 needed insulin injection, 3 were unable to start immediately and 4 opted to be out of the study due to not accustom to the taste of the breakfast supplement). A total of 71 patients met the inclusion criteria and were willing to participate in our study at the end (Figure 1). Recruitment started from 20 June 2013 with the first participant being found on 2 July to the last visit on 30 October 2013. The inclusion criteria were as follows: subjects had to be diagnosed with T2DM according to the American Diabetes Association (ADA) criteria for the diagnosis of diabetes [11], between 18 and 75 years old, and had a BMI of >18.5 kg/m^2^ and <35 kg/m^2^. If the patients were taking medication for a condition, such as anti-hypertensive medication, lipid-lowering medication, thyroid medication or hormone therapy, they had to have been on a consistent dosage for at least two months prior to the screening visit. Exclusion criteria included use of exogenous insulin for glucose control, mental disorders, cancer, cirrhosis, renal disease, hepatic disease and having a significant cardiovascular event less than six months prior to the screening visit. Subjects who were known to be allergic or intolerant to any ingredient of the study supplements were also excluded.

### 2.2. Intervention Content

Participants in treatment group were offered multi-nutrient supplements, which were provided by the LEHEL Company, Guangzhou, China. The main ingredients in the powdered supplement were rice, soybean, oat dietary fiber, resistant starch, bitter gourd, multi-vitamins and multi-minerals (Supplementary Table S1). There was a high content of dietary fiber (8.0 g per 100 g supplement) in the multi-nutrient supplement. We estimated the glycemic index of the supplements prior to the study using 5 male and 5 female healthy volunteers that were 22.1 ± 0.6 years old with a Body mass index (BMI) of 20.2 ± 1.2 kg/m^2^. All the volunteers were fed with 95.2 g nutrition product (contains 50 g carbohydrate) in the morning after overnight fasting and 50 g glucose 7 days later with an empty stomach as well [12]. The GI value was 33 (95% confidence interval (CI), 22 to 43), as calculated using an indefinite integral (Supplementary Figure S1).

### 2.3. Study Design

The study was designed as a randomized, open label, interventional study on patients with T2DM. The patients were classified by sex and hemoglobin A1c (HbA1c) values per the suggestion of the American Diabetes Association concerning ideal HbA1c goals (HbA1c value ≤ 7.0% of total hemoglobin) [13]. In order to attract more subjects to participate, an unequal randomization ratio was used in the study [14]. Participants were randomly assigned at a 2:1 ratio to either the treatment group consuming 75 g of the low glycemic index multi-nutrient supplement (provides 346 kcal energy) in place of breakfast or the control group consuming a healthy breakfast for 12 weeks. The breakfast in local tradition is much simpler than lunch and dinner, so breakfast was selected for replacement. The amount of supplement was selected due to our previous dietary survey in T2DM patients showing that the energy intake of breakfast is about 350 kcal [15]. The multi-nutrient supplement was steeped in 300 mL hot water and orally ingested. All participants were compensated for participating in the study. Neither the investigators nor the participants were blinded. The goal of this study was to evaluate the potential effect of the multi-nutrient supplement as a low GI breakfast on glycemic control in T2DM patients.

After confirming their participation in the study, all the study subjects signed an informed consent form. Then physical measurements, including height, weight, waist circumference and blood pressure were performed. Fasting blood samples were collected at week 0 (baseline) and week 12 and stored at −80 °C. Furthermore, individual food intake was recorded by 3-day and 24-h recalls, which consisted of two weekdays and one weekend day at week 0 and week 12. The breakfast replacement group got a two weeks supply of the supplement starting from week 0. All the participants were followed up at weeks 0, 2, 4, 6, 8, 10 and 12 during the study and had their 2 h blood sugar measured with a glucometer (Roche Diagnostics, Basel, Switzerland) after breakfast. At each 2-week appointment, the breakfast replacement group returned the empty packages and gained the amount for the next interval. The patient compliance was evaluated by counting their empty packages and the results were included in data analysis if they used more than 80% of their supplements. All the participants received diabetic health education organized by nutritionists every 2 weeks, including lifestyle and diet suggestions. We had a recommended food list for the patients in the diet suggestions, which mainly composed of low GI foods (Supplementary Table S2), and more importantly, they were taught the idea of having “balanced diet, and splitting up and little meals at each”. Taking the risk of hypoglycemia in consideration, snacking was allowed between meals, and skipping breakfast was not recommended. Anyone who had elevated blood sugar with a postprandial blood glucose >11.1 mmol/L for two consecutive 2-week follow ups induced by an unknown reason was recommended to quit the study and increase the hypoglycemic agent in accordance with their doctor’s advice.

The study was in accordance with the guidelines from the Declaration of Helsinki, and all procedures involving human participants were approved by the Research Ethics Board of Sun Yat-Sen University. This trial was registered at clinicaltrials.gov as NCT01940302.

### 2.4. Data Collection

Trained interviewers administered a questionnaire to collect information on the demographic characteristics, duration of diabetes, age at onset of diabetes, medications being taken, and frequency of smoking and drinking of participants. As to somatometry, at week 0 and week 12, weight, height, and waist circumference of the patients were obtained. Standing height was measured using a digital stadiometer (LEKA, Zhengzhou, China) with a fixed vertical backboard and an adjustable headpiece. And weight in an examination gown was measured on a digital scale. BMI was calculated as weight in kilograms divided by height in meters squared. Waist circumference at the end of a normal exhalation was measured to the nearest 0.1 cm with a measuring tape positioned just above the uppermost lateral border of the ilium. Seated blood pressure was measured in triplicate with an automatic sphygmomanometer (EW3106, Panasonic, Osaka, Japan). A family history of diabetes was defined as positive if any first- or second-degree relatives had T2DM. Smoking was defined as at least 1 cigarette per day for at least 6 months, and alcohol use was defined as drinking alcohol at least once a week for more than 6 months [16]. The fat mass was estimated as the percentage of body weight using bioelectrical impedance analysis (UM-41, TANITA, Dongguan, China).

The diet surveys were performed as previously described with 3-day dietary recalls [17]. The interviewers were uniformly trained and were familiar with local food species and price.

An International Physical Activity Questionnaires (IPAQ) short form was used to determine the physical activity level of participants and a Mini Nutritional Assessment (MNA) scale was used to determine nutrition status [18,19].

### 2.5. Biochemical and Dietary Analyses

Whole blood collected in heparin sodium anticoagulation tubes was analyzed within 24 h of collection to determine liver function, renal function, blood lipid levels, blood glucose levels and HbA1c values. Analysis was done by the hospital routine analytical laboratory using an automatic biochemical analyzer (HITACHI 7060, Tokyo, Japan). The EDTA anticoagulant blood plasma levels of insulin were measured with ELISA kits (Millipore, Billerica, MA, USA). The insulin resistance was evaluated through homeostasis model assessment (HOMA-IR) and calculated as [fasting insulin (mU/L) × fasting glucose (mmol/L)]/22.5 [20].

Diet records were analyzed using the nutrition software Automatic Catering 10.0 (Jiandian Inc., Beijing, China).

### 2.6. Statistical Analyses

Results were expressed as mean ± SD, median ± quartile or 95% CI. The questionnaires were double recorded into a computer with EpiData software [21] and statistical analyses were conducted with SPSS 17.0 (IBM-SPSS, Chicago, IL, USA). Differences in demographic characteristics were calculated using χ^2^ test for categorical variables and nonparametric Wilcoxon test for continuous variables. Paired sample Student’s *t* test was used to compare the baseline and values at week 12 within the control group or treatment group, while independent sample Student’s *t* test was used to compare between the control and treatment groups. All the statistical tests were based on the two-tailed hypothesis and the significance level was defined as *p* < 0.05.

## 3. Results

### 3.1. Characteristics of the Patients

A total of 54 participants were spitted into 18 in the control group and 36 in the breakfast replacement group and the 12-week study as summarized in Figure 1. There were no significant differences between the two groups in terms of baseline demographic characteristics, MNA scores, physical activity, blood lipid levels, blood pressure or anthropometry. 32% of participants in the treatment group and 18% in the control group, respectively, used antihyperglycemic medications (*p* = 0.289), and little difference was found in sulfonylurea use between the two groups (Table 1).

**Table 1:** Baseline characteristics of study participants.

Characteristics	Participants, No. (%) ^†^		*p*
	Breakfast Replacement (*n* = 36)	Control (*n* = 18)	
Age, year	56.7 ± 8.6	54.5 ± 10.1	0.410
Gender			
Male	22 (61.1)	11 (61.1)	1.000
Female	14 (38.9)	7 (38.9)	
Race			
Han	35 (97.2)	17 (94.4)	1.000
Yao	1 (2.8)	1 (5.6)	
Weight, kg	64.3 ± 9.1	61.2 ± 11.4	0.270
Body Mass Index	24.6 ± 2.6	23.7 ± 2.9	0.234
Family History ^§^	14 (38.9)	8 (44.4)	0.695
Educational level			
High School	20 (55.6)	10 (55.6)	1.000
Below	16 (44.4)	8 (44.4)	
Current Smoker	19 (52.8)	7 (38.9)	0.336
Current Drinker	7 (19.4)	3 (16.7)	1.000
Physical Activity			
Low	8 (22.2)	2 (11.1)	0.448
Moderate	21 (58.3)	12 (66.7)	
High	7 (19.4)	4 (22.2)	
HbA1c Value, % of Total Hemoglobin	6.7 ± 0.9	6.5 ± 0.6	0.352
≤7.0%	26 (72.2)	14 (77.8)	0.511
>7.0%	10 (27.8)	4 (22.2)	
Duration of T2DM, year	5.3 ± 4.4	4.4 ± 4.4	0.527
Antihyperglycemic Medications	32 (88.9)	18 (100)	0.289
Biguanides	27 (75.0)	11 (61.1)	0.292
Sulfonylurea	11 (30.6)	11 (61.1)	0.031
Glinides	9 (25.0)	4 (22.2)	1.000
α-glucosidase Inhibitors	6 (16.7)	1 (5.6)	0.403
Thiazolidinedione	1 (2.8)	3 (16.7)	0.103
DPP-IV Inhibitor	0 (0)	0 (0)	
Glp-1 Receptor Agonist	0 (0)	0 (0)	
Cholesterol-Lowering Medications	14 (38.9)	7 (38.9)	1.000
Blood Pressure Medications	12 (33.3)	8 (44.4)	0.348
Hyperuricemia Medications	0 (0)	0 (0)	
Renal Protection Medications	1 (2.8)	0 (0)	1.000
MNA Score, Median ± Quartile ^‡^	26.0 ± 1.5	26.0 ± 2.0	0.485
Well-Nourished	34 (94.4)	16 (88.9)	0.223
At Risk of Malnutrition	1 (2.7)	2 (11.1)	
Malnutrition	0 (0)	0 (0)	

Abbreviations: No., number. ^†^ Data are presented as number of patients with percentage in parentheses or mean ± SD, unless otherwise indicated; ^§^ A family history of diabetes was defined as positive if any first- or second-degree relatives had T2DM; ^‡^ There was a missing value in treatment group.

### 3.2. Diet and Physical Activity

According to the count of the recalled empty packages at every visit, compliance was very good in participants who completed the study. The rate of supplement intake in breakfast replacement group was 90.3%. Taking the study design into consideration, we appraised the diet of the participants at baseline and week 12 in each group (Table 2). There were no significant differences in energy and protein intake between baseline and week 12 in both groups. Also, no significant differences were found for caloric intake or energy changes for breakfast (breakfast calories: *p* = 0.922 and 0.488 and breakfast energy percent: *p* = 0.056 and *p* = 0.307 in treatment and control groups, respectively). However, both the control and treatment groups had significant differences in fat consumption between baseline and week 12 with a *p* = 0.009 and *p* = 0.017, respectively. When comparing the nutrients’ calorie percentages, both groups had a higher percent of fat (95% CI, 6.0% to 12.5%, *p* < 0.001 in treatment group, and 95% CI, 3.1% to 12.4%, *p* = 0.003 in control group) and lower percent of carbohydrates (95% CI, −12.2% to −5.9%, *p* < 0.001 in treatment group, and 95% CI, −14.5% to −4.2%, *p* = 0.001 in the control group) at week 12 compared to baseline.

When assessing nutrient intake at breakfast (Table 2), there were increased fat and decreased carbohydrate contents both in the control and treatment group (*p* = 0.013 and 0.006 in control group, *p* < 0.001 and *p* = 0.006 in treatment group, respectively). But no differences were found between groups with *p* = 0.492 and *p* = 0.562 of fat and carbohydrate intake of breakfast. Importantly, the dietary fiber level was higher in the breakfast replacement group at week 12 compared to baseline (95% CI, 2.2 to 4.3, *p* < 0.001) with a significant difference between the groups (*p* < 0.001).

As physical activity is a major influencing factor on glycemic control, the level of physical activity was estimated as well. There were no significant differences between groups at the baseline or the end of the study (*p* = 0.448 at baseline and *p* = 0.808 at week 12).

### 3.3. Glycemic Control and Somatometry

The breakfast replacement group had no significant increase in fasting blood glucose (FBG) at week 12, while the FBG in control group increased by 1.4 mmol/L (95% CI for change, 0.8 to 1.9 mmol/L, *p* < 0.001). When assessing short-term glycemic control indicators, glycated serum protein (GSP) decreased in the treatment group by −14.5 μmol/L (95% CI was −23.9 to −5.1 μmol/L, *p* = 0.004). In terms of long-term glycemic control, the HbA1c of participants taking the breakfast supplement decreased by −0.2% (95% CI, −0.38% to −0.07%, *p* = 0.004), while the HbA1c of the control group increased by 0.3% (95% CI, 0.1% to 0.5%, *p* = 0.005). The treatment difference was also significant between the two groups (*p* < 0.001). No significant differences in fasting insulin concentrations were found. In addition, we estimated the HOMA-IR of both groups and discovered that the HOMA-IR of the control group had risen by 0.7 (95% CI, 0.2 to 1.3, *p* = 0.01) with a significant difference between the groups (*p* = 0.018) (Table 3).

**Table 2:** Dietary intake of control and treatment groups at baseline and week 12 ^†^ (Mean ± SD).

Items	Breakfast Replacement (*n* = 36)			Control (*n* = 18)			*p* ^§^
	Baseline	Week 12	Mean Change (95% CI) ^‡^	Baseline	Week 12	Mean Change (95% CI) ^‡^	
Total Day							
Energy (kcal)	1567.1 ± 512.9	1445.6 ± 400.9	−121.4 (−290.5, 47.6)	1425.8 ± 352.5	1523.2 ± 550.2	97.4 (−114.0, 308.8)	0.118
Protein (g)	57.8 ± 22.4	79.8 ± 93.2	22.1 (−8.3, 52.5)	54.7 ± 14.2	55.4 ± 18.4	19.5 (−9.0, 10.5)	0.327
Protein (%)	14.6 ± 2.4	24.3 ± 38.5	9.7 (−3.3, 22.6)	15.5 ± 2.7	15.1 ± 3.7	−0.4 (−2.4, 1.6)	0.272
Fat (g)	53.1 ± 23.9	63.2 ± 23.3 *	10.2 (1.9, 18.4)	49.3 ± 18.1	63.9 ± 24.4 **	14.6 (4.1, 25.1)	0.515
Fat (%)	30.5 ± 7.5	39.7 ± 9.8 **	9.3 (6.0, 12.5)	31.0 ± 8.1	38.8 ± 8.9 **	7.8 (3.1, 12.4)	0.593
Carbohydrate (g)	213.2 ± 73.6	164.6 ± 58.2 **	−48.6 (−74.5, −22.7)	184.1 ± 53.9	166.7 ± 81.3 **	−17.4 (−49.2, 14.5)	0.142
Carbohydrate (%)	54.6 ± 8.1	45.6 ± 8.8 **	−9.0 (−12.2, −5.9)	52.1 ± 9.4	42.7 ± 8.0 **	−9.4 (−14.5, −4.2)	0.907
Breakfast							
Energy (kcal)	404.9 ± 180.0	408.0 ± 97.6	3.1 (−61.4, 67.6)	420.5 ± 144.8	391.4 ± 145.3	−29.1 (−115.6, 57.4)	0.55
% of Total Energy	26.2 ± 8.4	30.0 ± 10.0	3.8 (−0.1, 7.7)	29.5 ± 7.8	26.4 ± 7.9	−3.1 (−9.3, 3.1)	0.049
Protein (g)	12.9 ± 5.5	14.8 ± 4.0	1.9 (−0.6, 4.4)	14.1 ± 4.9	13.1 ± 5.0	−1.0 (−4.0, 2.0)	0.158
Fat (g)	10.3 ± 7.9	17.1 ± 6.0 **	6.9 (3.8, 9.9)	8.0 ± 3.9	13.2 ± 7.6 *	5.1 (1.2, 9.1)	0.492
Carbohydrate (g)	67.2 ± 33.2	51.3 ± 15.7 **	−15.9 (−26.9, −4.9)	77.5 ± 32.8	56.4 ± 23.5 **	−21.2 (−35.2, −7.1)	0.562
Dietary Fiber (g)	3.0 ± 2.7	6.2 ± 2.4 **	3.2 (2.2, 4.3)	2.7 ± 1.3	2.0 ± 1.6	−0.7 (−1.5, 0.1)	<0.001
Lunch							
Energy (kcal)	553.9 ± 205.8	485.0 ± 163.4 *	−68.9 (−129.7, −8.1)	509.0 ± 162.8	516.9 ± 214.5	7.9 (−89.7, 105.4)	0.157
% of Total Energy	35.3 ± 5.2	33.7 ± 6.4	−1.5 (−3.8, 0.7)	35.5 ± 5.4	34.1 ± 7.5	−1.4 (−5.6, 2.9)	0.915
Dinner							
Energy (kcal)	608.3 ± 252.5	487.0 ± 172.9 **	−121.3 (−207.5, −35.0)	496.2 ± 124.2	599.1 ± 311.1	102.9 (−54.0, 259.7)	0.007
% of Total Energy	38.6 ± 7.4	34.1 ± 8.9 *	−4.5 (−8.6, −4.3)	35.0 ± 3.9	42.0 ± 24.4	7.0 (−6.2, 20.2)	0.032

^†^ Dietary measures that were obtained at week 0 represent baseline and at week 12 represent the end of the study; ^‡^ Mean changes were calculated by subtracting the baseline values from the 12 week values of each group; ^§^ *p* values for mean changes between control and treatment group at week 12 from baseline were analyzed by independent sample Student’s *t* tests; Percentages represent the percentage of total calories per day. Significant changes (* *p* < 0.05, ** *p* < 0.01; Paired Student’s *t* test) within group from baseline to week 12.

**Table 3:** Biochemistry and somatometry of control and treatment groups at baseline and week 12 ^†^ (Mean ± SD).

Characteristics	Breakfast Replacement (*n* = 36)			Control (*n* = 18)			*p* ^§^
	Baseline	Week 12	Mean Change (95% CI) ^‡^	Baseline	Week 12	Mean Change (95% CI) ^‡^	
Glycemic Control							
FBG (mmol/L)	6.8 ± 1.6	7.0 ± 1.5	0.2 (−0.1, 0.6)	6.8 ± 1.1	8.1 ± 1.3 **	1.4 (0.8, 1.9)	0.001
GSP (μmol/L)	274.3 ± 62.7	259.8 ± 53.1 **	−14.5 (−23.9, −5.1)	280.1 ± 45.3	276.0 ± 53.7	−4.1 (−18.8, 10.7)	0.209
HbA1c (%)	6.7 ± 0.9	6.5 ± 0.8 **	−0.2 (−0.38, −0.07)	6.5 ± 0.6	6.8 ± 0.8 **	0.3 (0.1, 0.5)	<0.001
Insulin (μU/mL)	4.8 ± 2.7	4.7 ± 3.2	−0.1 (−1.0, 0.9)	5.3 ± 2.2	6.4 ± 2.6	1.1 (−0.1, 2.3)	0.129
HOMA-IR	1.5 ± 1.0	1.5 ± 1.1	0.04 (−0.3, 0.4)	1.7 ± 0.8	2.4 ± 1.0 *	0.7 (0.2, 1.3)	0.018
Physical Activity, No. (%) ^¶^							
Low	8 (22.2)	7 (28)		2 (11.1)	0 (0)		
Moderate	21 (58.3)	8 (32)		12 (66.7)	14 (77.8)		
High	7 (19.4)	10 (40)		4 (22.2)	4 (22.2)		
Somatometry							
Weight (kg)	64.4 ± 9.1	63.9 ± 9.6	−0.4 (−1.7, 0.9)	61.2 ± 11.4	62.5 ± 11.2 **	1.3 (0.4, 2.3)	0.07
BMI (kg/m^2^)	24.6 ± 2.6	24.4 ± 2.4	−0.2 (−0.7, 0.2)	23.7 ± 2.9	24.2 ± 2.8 **	0.5 (0.2, 0.9)	0.032
Waistline (cm)	86.3 ± 7.3	84.1 ± 7.4 **	−2.2 (−3.4, −1.0)	82.6 ± 9.3	83.2 ± 8.9	0.6 (−1.8, 2.9)	0.021
Hipline (cm)	95.1 ± 5.3	94.5 ± 5.8	−0.6 (−1.5, 0.3)	92.9 ± 6.4	92.9 ± 6.6	−0.03 (−1.4, 1.4)	0.475
WHR	0.91 ± 0.04	0.89 ± 0.05 **	−0.02 (−0.03, −0.01)	0.89 ± 0.07	0.90 ± 0.07	0.01 (−.02, 0.03)	0.037
SBP (mmHg)	124.1 ± 14.1	127.7 ± 14.3	3.5 (−2.0, 9.1)	120.7 ± 15.3	134.2 ± 22.2 *	13.5 (3.5, 23.5)	0.058
DBP (mmHg)	79.6 ± 7.7	80.6 ± 8.2	0.9 (−2.1, 4.0)	76.4 ± 7.3	83.2 ± 9.9 **	6.8 (2.7, 10.9)	0.026
MAP (mmHg)	94.5 ± 9.3	95.9 ± 9.7	1.8 (−1.7, 5.3)	91.1 ± 9.3	102.3 ± 13.1 **	9.0 (3.6, 14.5)	0.026
Nutrition Status							
MNA Score	26.0 ± 1.5	26.0 ± 3	−0.4 (−1.2, 0.4)	26.0 ± 2.0	26.8 ± 2.3	0.0 (−0.9, 0.9)	0.412
Body Fat (%) ^††^	28.0 ± 7.0	28.2 ± 6.7	0.2 (−0.4, 0.8)	26.7 ± 6.6	28.2 ± 6.7 **	1.5 (0.6, 2.4)	0.015
Body Water (%) ^††^	52.5 ± 5.1	52.4 ± 4.9	−0.1 (−0.6, 0.3)	53.7 ± 4.8	52.6 ± 4.9 **	−1.1 (−1.8, −0.5)	0.013
Total Plasma Protein (g/L)	74.9 ± 4.6	76.8 ± 4.5 **	1.9 (0.9, 3.0)	75.7 ± 4.4	77.4 ± 4.5	1.7 (−0.4, 3.8)	0.821
Plasma Lipid Levels							
TG (mmol/L)	1.9 ± 1.3	2.1 ± 2.6	0.2 (−0.3, 0.8)	2.9 ± 4.9	2.0 ± 2.4	−0.8 (−2.7, 1.0)	0.27
Cholesterol (mmol/L)	5.0 ± 1.0	5.1 ± 0.8	0.2 (−0.1, 0.4)	5.4 ± 1.5	5.3 ± 1.0	−0.1 (−0.6, 0.4)	0.276
HDL-C (mmol/L)	1.4 ± 0.4	1.4 ± 0.4	0.01 (−0.1, 0.1)	1.4 ± 0.3	1.5 ± 0.4	0.1 (−0.1, 0.3)	0.279
LDL-C (mmol/L)	3.1 ± 0.8	3.0 ± 0.4	−0.1 (−0.3, 0.1)	3.3 ± 1.1	3.1 ± 0.6	−0.2 (−0.6, 0.3)	0.748
ApoA1 (g/L)	1.2 ± 0.2	1.3 ± 0.2	0.04 (−0.03, 0.11)	1.2 ± 0.2	1.3 ± 0.2	0.1 (−0.01, 0.22)	0.287
ApoB (g/L)	0.9 ± 0.1	0.9 ± 0.1	0.01 (−0.03, 0.05)	0.9 ± 0.2	1.0 ± 0.2 *	0.1 (0.01, 0.16)	0.049
Hepatorenal functions							
AST (IU/L)	22.7 ± 5.0	24.0 ± 5.5	1.3 (−0.4, 3.0)	26.7 ± 10.7	32.7 ± 12.4	5.9 (−0.1, 12.0)	0.138
ALT (IU/L)	23.6 ± 13.4	22.2 ± 9.2	−1.4 (−5.6, 2.9)	25.6 ± 15.0	25.4 ± 12.1	−0.1 (−7.1, 6.9)	0.738
Total bilirubin (μmol/L)	14.4 ± 3.1	14.3 ± 5.2	−0.2 (−1.9, 1.6)	14.1 ± 4.4	15.8 ± 6.6	1.7 (−0.4, 3.8)	0.185
Urea nitrogen (mmol/l)	5.4 ± 1.4	5.8 ± 1.4 *	0.4 (0.02, 0.79)	5.6 ± 1.6	6.6 ± 2.6 *	1.0 (0.2, 1.8)	0.118
Creatinine (μmol/L)	87.4 ± 13.7	81.3 ± 11.0 **	−6.1 (−9.3, −3.0)	87.8 ± 14.6	84.9 ± 14.7	−2.9 (−6.0, 0.3)	0.138
Uric acid (μmol/L)	344.3 ± 75.3	357.0 ± 78.9	12.7 (−8.3, 33.7)	368.5 ± 158.6	357.0 ± 111.1	−11.5 (−72.3, 49.4)	0.341

Abbreviations: ALT, alanine aminotransferase; ApoA1, Apolipoprotein A1; ApoB, Apolipoprotein B; AST, aspartate aminotransferase; BMI, body mass index, calculated as weight in kilograms divided by height in meters squared; DBP, diastolic blood pressure; FBG, fasting blood glucose; GSP, glycated serum protein; HbA1c, hemoglobin A1c; HOMA-IR, homeostasis model assessment–insulin resistance index, calculated as FBG multiplied by the FINS and then divided by 22.5; HDL-C, high density lipoprotein-cholesterol; LDL-C, low density lipoprotein-cholesterol; MAP, mean arterial pressure, calculated as 1/3 of SBP plus 2/3 of DBP; SBP, systolic blood pressure; TG, total triglyceride; WHR, waist hip ratio, calculated as waist circumference divided by the hip circumference in centimeters; ^†^ Data were obtained at week 0 to represent baseline and at week 12 to represent the end of the study; ^‡^ Mean changes for each group were calculated by subtracting the baseline from values at week 12; ^§^ *p* values for mean changes between control and treatment group at week 12 from baseline were analyzed by independent sample Student’s *t* test; ^¶^ Physical activity was expressed as the percentage of the total number of patients, and a missing value was found in treatment group at week 12; ^††^ % means the percentage of body weight; Significant changes (* *p* < 0.05, ** *p* < 0.01; paired Student’s *t* test) within group from baseline to week 12.

There was a statistically significant difference in BMI between the treatment and control groups (*p* = 0.032) due to the weight gain in the control group (mean change was 0.5; 95% CI was 0.2 to 0.9, *p* = 0.007). Not surprisingly, there was a discrepancy in the mean changes of WHR (*p* = 0.037). However, it was interesting to note that the blood pressure of the control group had increased at week 12 from the baseline (SBP, 95% CI, 3.5 to 23.5 mmHg, *p* = 0.012; DBP, 95% CI, 2.7 to 10.9 mmHg, *p* = 0.003; mean arterial pressure, MAP, 95% CI, 3.6 to 14.5, *p* = 0.003) (Table 3).

We also tested whether there was a relationship between the changes in weight and HbA1c, however, there was no significant correlation found (the spearman correlation coefficients were 0.289 and −0.234, *p* = 0.088 and *p* = 0.351 in the treatment and control groups, respectively).

### 3.4. Plasma Lipid Levels

No significant changes in plasma triglycerides, total cholesterol, low-density lipoprotein-cholesterol, high-density lipoprotein-cholesterol or apolipoprotein A1 were found. The apolipoprotein B levels in the control group had increased by 0.1 g/L (95% CI, 0.01 to 0.16, *p* = 0.034) at week 12 compared to baseline (Table 3).

### 3.5. Hepatorenal Functions

The plasma total bilirubin, aspartate and alanine aminotransferase activities didn’t change a lot during the study. However, both the control and treatment groups had increased blood urea nitrogen levels at week 12 compared to baseline (Table 3).

### 3.6. Nutrition Status

Both groups had no significant changes in MNA score (*p* = 0.223 in the treatment group and *p* = 0.435 in the control group). However, participants in control group, who ate a normal breakfast, displayed a significant increase in fat percentage and significant decrease in water percentage (*p* = 0.002 for both). There were also significant differences between the groups (*p* = 0.015 for fat percentage and 0.013 for water percentage, respectively) (Table 3).

## 4. Discussion

From this intervention study, we found that taking a low GI multi-nutrient supplement in place of breakfast lowered T2DM patient glycated serum protein, HbA1c, increased their total plasma proteins, and helped maintain their FBG, HOMA-IR, blood pressure and body weight. Our findings provide further support for the use of meal replacements as a nutritional strategy for weight loss and glycemic control in type 2 diabetes. As we know, lifestyle greatly affected glycemic control [22]. In this study, all the participants received diabetic health education every two weeks, which may have minimized the differences caused by physical activity and diet, except those caused by our interventions. The study results support this conclusion.

In order to ensure the low GI and energy supply of the treatment supplement, we chose rice and low GI soybean as the primary raw materials with oat dietary fiber and bitter gourd added [20,23]. 75 g of the multi-nutrient supplement provides about 349 kcal of energy, which was 17% of the nutrient reference value (NRV) and within the normal range of breakfast energy intake [24]. According to the estimated glycemic index test, the GI of the multi-nutrient supplement was 33, thus putting it into the low GI food category [25]. In this study, no differences in energy intake between baseline and week 12 were observed for both groups, as well as between the groups, thus ruling out any changes that could be caused by energy intake differences. The breakfast nutrient analysis showed that the treatment group had increased dietary fiber intake (mean change 3.2 g/day), which may help with glycemic control in this group [26].

In terms of blood glucose control, the HbA1c had a 0.2% absolute reduction at week 12 from the baseline in the treatment group and 0.5% relative reduction between the treatment and control groups. This reduction coincides with the proposal by the US Food and Drug Administration that a reduction of 0.3% to 0.4% in HbA1c values is therapeutically meaningful [27]. Furthermore, there was a significant difference in HOMA-IR changes between the groups (*p* = 0.018), suggesting an improvement of insulin resistance in the treatment group. However, increases in both HbA1c and HOMA-IR observed in the control group were unexpected. Upon further consideration, we realized that there may be a correlation between the timing of the research period and this phenomenon. There are many traditional festivals in China, and the Moon Festival and the National Day fell during our June to October study. The 0.3% increase in HbA1c in the control group was similar to a report on the glycemic control during holiday time in T2DM patients in China. Seasonal changes are also thought to influence preprandial glucose and HbA1c [28,29].

In terms of somatometry, our study found a lower BMI and WHR in the treatment group at week 12 compared to baseline (*p* = 0.032 and *p* = 0.037, respectively). This finding indicates that low GI breakfast replacements are an effective way to promote weight loss in T2DM patients in the absence of reduced energy intake. T2DM typically accompanies weight gain and obesity, and the control of obesity in these patients is a popular method of treating diabetes [30,31]. The breakfast replacement supplement may have helped glycemic control in this way. Also, this is the first trial to suggest a BP maintenance effect of low GI breakfast replacements.

Because dietary restriction can lead to malnutrition [32,33], we also focused on the nutritional status of the T2DM patients. The MNA scores showed that the T2DM patients in both the control and treatment groups were well-nourished (MNA score = 26.0 in both groups at baseline), and the supplement in place of breakfast had no effect on the nutritional status of the patients (95% CI of mean change was −1.2 to 0.4, *p* = 0.23).

However, the weaknesses of this study should also be taken into account. First, the method used for GI measuring in our study was not based on the guidelines fixed by the International Organization for Standardization (ISO) [34], combining with the small simple size (*n* = 10), this may cause a high degree of variation (standard error of the mean, SEM = 9.1). Secondly, there was a high dropout rate in our study (23.4% of the treatment group and 25.0% of the control group). In our opinion, one reason for patients in the treatment group to quit the study was the bitter taste of the supplement caused by the bitter gourd additive, which makes taste improvement of the product a priority. An in-depth investigation should be conducted concerning the control group dropout rate and how to prevent this. Also, due to budgetary constraints, our sample size was limited. Furthermore, the effect of α-glucosidase inhibitors on the results of this study is unknown, because the participants taking these inhibitors were not excluded [35]. Fortunately, good compliance by study participants was ensured by frequent visits and similar medication use between the groups and this may have minimized the disadvantages described above.

Despite the areas where our study needs to be improved, our findings link a low GI breakfast replacement to improved glycemic and weight control in T2DM patients. This is particularly important because the prevalence of type 2 diabetes has been increasing rapidly worldwide. Furthermore, dietary intake plays a crucial role in glucose control [1,4], but studies on the compliance of dietary therapy found only a short-term effect even after self-management training [36]. Therefore, a simpler and more effective way of treating T2DM needs to be found. In combination with stable blood pressure and maintenance of nutrition status, our study puts forward a treatment possibility for controlling blood glucose levels that is easily accepted by type 2 diabetes patients.

## 5. Conclusions

These data suggest that measurable health advantages are associated with a low GI multi-nutrient breakfast replacement in patients with T2DM. This simple dietary strategy to control blood glucose and body weight merits further research and the consideration of health practitioners.

## Supplementary Files

Supplementary File 1
