# Supplementary material for: Taking a Low Glycemic Index Multi-Nutrient Supplement as Breakfast Improves Glycemic Control in Patients with Type 2 Diabetes Mellitus: A Randomized Controlled Trial
Source: Nutrients. 2014 Dec 10;6(12):5740–55. doi: 10.3390/nu6125740 (PMC4276995; doi:10.3390/nu6125740)
Supplement: Supplementary File 1 [file nutrients-06-05740-s001.docx]

Supplementary information

**Table S1.** Components of the multi-nutrient supplement.

| **Items** | **Per 75 g** | **Items** | **Per 75 g** |
| --- | --- | --- | --- |
| Energy | 1461.0 KJ (346.5 Kcal) | Vitamin C | 18.8 mg |
| Protein | 13.1 g | Nicotinic acid | 2.8 mg |
| Fat | 15.4 g | Potassium | 375.0 mg |
| Carbohydrate | 39.4 g | Magnesium | 66.0 mg |
| Dietary Fiber | 6.0 g | Calcium | 187.5 mg |
| Sodium | 0.3 g | Iron | 3.8 mg |
| Vitamin A | 150.0 μg RE | Zinc | 4.9 mg |
| Vitamin B1 | 0.2 mg | Cuprum | 0.1 mg |
| Vitamin B2 | 0.3 mg | Manganese | 0.1 mg |
| Vitamin B6 | 0.2 mg | Chromium | 56.3 μg |

Abbreviation: RE, retinol equivalent

**Figure S1.** Plasma glucose (**a**) and insulin (**b**) following consumption of 50 g glucose or 95.2 g multi-nutrient supplement. Statistical significance between the values for the supplement and reference food is indicated by ***** for *p* < 0.05 and ******* for *p* < 0.001.


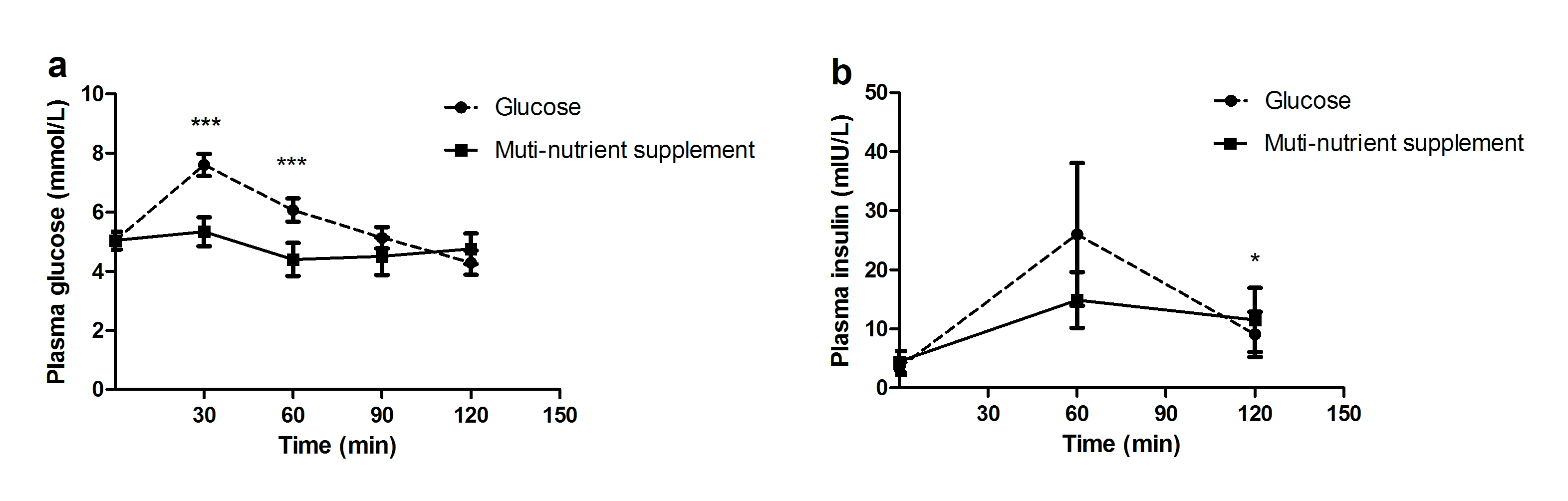


**Table S2.** Recommended foods for type 2 diabetic patients.

| **Recommended Food for Breakfast** | **Examples** |
| --- | --- |
| Staple Food | Wholemeal, oatmeal, buckwheat, dumplings, teamed stuffed bun |
| Proteins | Pork, chicken, beef, fish, shrimp, sausages, bacon, duck, soybean, tofu, peanut, eggs, milk, yoghourt |
| Vegetables/Fruits | Lotus root, tomato, fungus, mushroom, spinach, onion, bamboo shoots, green pepper, eggplant, cucumber, kelp, kidney bean, bitter gourd, ternip, lettuce, peach, apple, kiwi, cherry, strawberry, lemon |
| Oil | Olive oil, colza oil, camellia oil |
| Samples of Food Combination in Breakfast (1800 Kcal/D, 20% Breakfast Energy) | Tomato 50 g, an egg (60 g), noodles 100 g;  porridge 25 g, steamed buns 60 g, an egg (60 g), sausage 35 g |

© 2014 by the authors; licensee MDPI, Basel, Switzerland. This article is an open access article distributed under the terms and conditions of the Creative Commons Attribution license (http://creativecommons.org/licenses/by/4.0/).
